# *catena*-Poly[[(1,10-phenanthroline)copper(II)]-μ-oxalato]

**DOI:** 10.1107/S1600536810035440

**Published:** 2010-09-08

**Authors:** Jun Wang, Yong Hou, Zhi-li Fang

**Affiliations:** a Zhongshan Polytechnic, Zhongshan, Guangdong 528404, People’s Republic of China; b School of Basic Science, East China Jiaotong University, Nanchang 330013, People’s Republic of China

## Abstract

In the title coordination polymer, [Cu(C_2_O_4_)(C_12_H_8_N_2_)]_*n*_, the Cu^II^ atom is six-coordinated by four O atoms from two oxalate ligands and two N atoms from one 1,10-phenanthroline (phen) ligand in a distorted octa­hedral coordination geometry. The oxalate anions act as bis-bidentate ligands, bridging the Cu–phen units in zigzag chains extending parallel to [100]. Inter­chain C—H⋯O hydrogen bonding and π–π stacking inter­actions [centroid–centroid distance = 3.7439 (17) Å] assemble neighboring chains, forming a three-dimensional supra­molecular network.

## Related literature

For the topologies and potential applications as functional materials of metal coordination polymers, see: Benneli & Gatteschi (2002 ▶); Qin *et al.* (2005 ▶); Qiu *et al.* (2007 ▶).
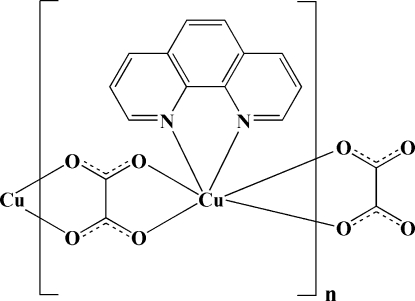


## Experimental

### 

#### Crystal data

[Cu(C_2_O_4_)(C_12_H_8_N_2_)]
*M* *_r_* = 331.76
Orthorhombic, 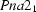

*a* = 9.1445 (8) Å
*b* = 10.1443 (9) Å
*c* = 13.3294 (11) Å
*V* = 1236.50 (18) Å^3^
*Z* = 4
Mo *K*α radiation
μ = 1.78 mm^−1^
*T* = 298 K
0.42 × 0.35 × 0.29 mm

#### Data collection

Bruker APEXII CCD area-detector diffractometer
Absorption correction: multi-scan (*SADABS*; Sheldrick, 2008 ▶) *T*_min_ = 0.544, *T*_max_ = 0.612
6811 measured reflections
2618 independent reflections
2373 reflections with *I* > 2σ(*I*)
*R*_int_ = 0.021

#### Refinement

*R*[*F* ^2^ > 2σ(*F* ^2^)] = 0.024
*wR*(*F* ^2^) = 0.059
*S* = 1.04
2618 reflections
190 parameters
1 restraint
H-atom parameters constrained
Δρ_max_ = 0.29 e Å^−3^
Δρ_min_ = −0.30 e Å^−3^
Absolute structure: Flack (1983 ▶), 1217 Friedel pairs
Flack parameter: 0.019 (14)

### 

Data collection: *APEX2* (Bruker, 2004 ▶); cell refinement: *SAINT* (Bruker, 2004 ▶); data reduction: *SAINT*; program(s) used to solve structure: *SHELXS97* (Sheldrick, 2008 ▶); program(s) used to refine structure: *SHELXL97* (Sheldrick, 2008 ▶); molecular graphics: *XP* in *SHELXTL* (Sheldrick, 2008 ▶); software used to prepare material for publication: *SHELXTL*.

## Supplementary Material

Crystal structure: contains datablocks I, global. DOI: 10.1107/S1600536810035440/zl2304sup1.cif

Structure factors: contains datablocks I. DOI: 10.1107/S1600536810035440/zl2304Isup2.hkl

Additional supplementary materials:  crystallographic information; 3D view; checkCIF report

## Figures and Tables

**Table 1:** Hydrogen-bond geometry (Å, °)

*D*—H⋯*A*	*D*—H	H⋯*A*	*D*⋯*A*	*D*—H⋯*A*
C11—H11⋯O4^i^	0.93	2.51	3.416 (4)	166
C9—H9⋯O1^ii^	0.93	2.49	3.160 (3)	129
C2—H2⋯O2^iii^	0.93	2.52	3.136 (3)	124
C1—H1⋯O4^iv^	0.93	2.56	3.072 (3)	115

Symmetry codes: (i) 
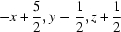
; (ii) 
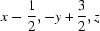
; (iii) 
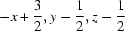
; (iv) 
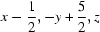
.

**Fig. 1.**
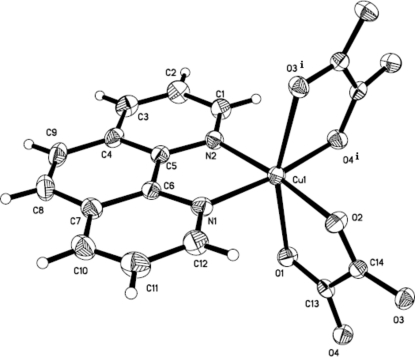
ORTEP represention of atom numbering diagram for the title complex, showing 30% probability displacement ellipsoids. Symmetry code: (i) –1/2 + x, 2.5–y, z.

**Fig. 2.**
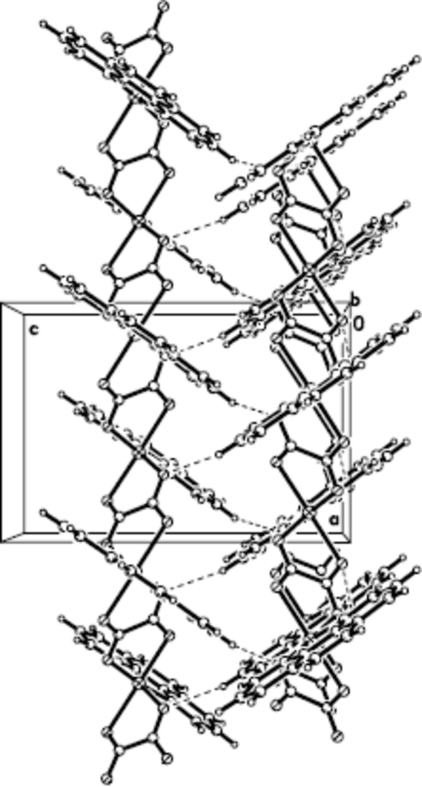
View of the three-dimensional structure of the title compound.

## supplementary crystallographic information

### Comment

The design and construction of metal coordination polymers based on metal ions and multifunctional bridging ligands is of great interest due to their intriguing topologies and potential applications as functional materials (Benneli & Gatteschi, 2002; Qiu *et al.*, 2007). Copper, with its variable coordination numbers and flexible coordination geometry, provides unique opportunities for the discovery of unusual networks in this interesting and challenging field (Qin *et al.*, 2005). We chose oxalate ligands as organic spacers since this rigid molecule has proven to be able to establish a bridge between metal centers. Herein, we present the structure of the title compound, [Cu(C_2_O_4_)(C_12_H_8_N_2_)]_n_.

The Cu^II^ atom exhibits a distorted octahedral configuration coordinated by four oxygen atoms from two oxalate ligands (Cu—O = 1.9753 (18)-2.3135 (18) Å) and two nitrogen atoms from one 1,10-phenanthroline ligand (Cu—N = 2.024 (2) and 2.049 (2) Å) (Fig. 1). The oxalate ligands bridge adjacent Cu-phen units to form a one-dimensional zigzag chain along the a-axis of the unit cell. The Cu—Cu separation is 5.529 (2) Å. Interchain π-π stacking interactions between phen ligands in neighboring chainslead to the formation of sheets of connected chains in the *ab*-plane. The centroid to centroid distances between neighboring 1,10-phenanthroline ligands is 3.7439 (17) Å [ring (C4-C9) to ring (N2, C1 to C5) (symmetry code: –1/2+*x*, 3/2–*y*, *z*)]. C–H···O hydrogen bonds interconnect these sheets to extend to a three-dimensional supramolecular network motif (Table 1; Fig. 2).

### Experimental

A sample of cupric acetate (0.0399 g, 0.20 mmol), oxalic acid (0.1015 g, 0.50 mmol), 1,10-phenanthroline (0.2523 g, 0.50 mmol), were added to water (10 ml). The resultant mixture was sealed in a 25 ml stainless steel reactor with a Teflon liner and kept under autogenous pressure at 413 K for 78 h, and then cooled to room temperature at a rate of 0.5 K/min. Colorless blocky crystals of the title compound suitable for single-crystal X-ray diffraction analyses formed in a yield of approximately 65%.

### Refinement

All H atoms were placed at calculated positions and were treated as riding on the parent C atoms with C—H = 0.93 Å, and with *U*_iso_(H) = 1.2 (C).

### Crystal data

**Table:**

[Cu(C_2_O_4_)(C_12_H_8_N_2_)]	*F*(000) = 668
*M**_r_* = 331.76	*D*_x_ = 1.782 Mg m^−^^3^
Orthorhombic, *P**n**a*2_1_	Mo *K*α radiation, λ = 0.71073 Å
Hall symbol: P 2c -2n	Cell parameters from 2618 reflections
*a* = 9.1445 (8) Å	θ = 2.5–27.0°
*b* = 10.1443 (9) Å	µ = 1.78 mm^−^^1^
*c* = 13.3294 (11) Å	*T* = 298 K
*V* = 1236.50 (18) Å^3^	Block, blue
*Z* = 4	0.42 × 0.35 × 0.29 mm

### Data collection

**Table:**

Bruker APEXII CCD area-detector diffractometer	2618 independent reflections
Radiation source: fine-focus sealed tube	2373 reflections with *I* > 2σ(*I*)
graphite	*R*_int_ = 0.021
φ and ω scan	θ_max_ = 27.0°, θ_min_ = 2.5°
Absorption correction: multi-scan (*SADABS*; Sheldrick, 2008)	*h* = −8→11
*T*_min_ = 0.544, *T*_max_ = 0.612	*k* = −10→12
6811 measured reflections	*l* = −16→15

### Refinement

**Table:**

Refinement on *F*^2^	Secondary atom site location: difference Fourier map
Least-squares matrix: full	Hydrogen site location: inferred from neighbouring sites
*R*[*F*^2^ > 2σ(*F*^2^)] = 0.024	H-atom parameters constrained
*wR*(*F*^2^) = 0.059	*w* = 1/[σ^2^(*F*_o_^2^) + (0.0289*P*)^2^] where *P* = (*F*_o_^2^ + 2*F*_c_^2^)/3
*S* = 1.04	(Δ/σ)_max_ = 0.001
2618 reflections	Δρ_max_ = 0.28 e Å^−^^3^
190 parameters	Δρ_min_ = −0.30 e Å^−^^3^
1 restraint	Absolute structure: Flack (1983), 1217 Friedel pairs
Primary atom site location: structure-invariant direct methods	Flack parameter: 0.019 (14)

### Special details

**Table:**

Geometry. All e.s.d.'s (except the e.s.d. in the dihedral angle between two l.s. planes) are estimated using the full covariance matrix. The cell e.s.d.'s are taken into account individually in the estimation of e.s.d.'s in distances, angles and torsion angles; correlations between e.s.d.'s in cell parameters are only used when they are defined by crystal symmetry. An approximate (isotropic) treatment of cell e.s.d.'s is used for estimating e.s.d.'s involving l.s. planes.
Refinement. Refinement of *F*^2^ against ALL reflections. The weighted *R*-factor *wR* and goodness of fit *S* are based on *F*^2^, conventional *R*-factors *R* are based on *F*, with *F* set to zero for negative *F*^2^. The threshold expression of *F*^2^ > σ(*F*^2^) is used only for calculating *R*-factors(gt) *etc*. and is not relevant to the choice of reflections for refinement. *R*-factors based on *F*^2^ are statistically about twice as large as those based on *F*, and *R*- factors based on ALL data will be even larger.

### Fractional atomic coordinates and isotropic or equivalent isotropic displacement parameters (Å^2^)

**Table:**

	*x*	*y*	*z*	*U*_iso_*/*U*_eq_	
C1	0.6938 (3)	0.9764 (3)	−0.04988 (19)	0.0407 (6)	
H1	0.6652	1.0617	−0.0662	0.049*	
Cu1	0.87241 (3)	1.09675 (2)	0.11815 (4)	0.03062 (9)	
N1	0.9474 (2)	0.9310 (2)	0.18929 (17)	0.0352 (5)	
O1	1.0814 (2)	1.12990 (18)	0.02682 (14)	0.0377 (4)	
C2	0.6422 (3)	0.8724 (3)	−0.1095 (2)	0.0486 (7)	
H2	0.5818	0.8883	−0.1643	0.058*	
N2	0.7802 (2)	0.9598 (2)	0.02762 (16)	0.0325 (4)	
O2	0.98329 (19)	1.21773 (18)	0.20589 (13)	0.0377 (4)	
C3	0.6829 (3)	0.7475 (3)	−0.0849 (2)	0.0472 (7)	
H3	0.6506	0.6770	−0.1237	0.057*	
O3	1.1635 (2)	1.36277 (17)	0.21108 (14)	0.0358 (4)	
C4	0.7727 (3)	0.7242 (2)	−0.0022 (2)	0.0386 (6)	
O4	1.2795 (2)	1.25638 (18)	0.04136 (14)	0.0376 (4)	
C5	0.8190 (3)	0.8351 (2)	0.05270 (19)	0.0321 (5)	
C6	0.9086 (2)	0.8194 (2)	0.13957 (17)	0.0307 (6)	
C7	0.9518 (3)	0.6918 (3)	0.1703 (2)	0.0404 (6)	
C8	0.9027 (3)	0.5816 (2)	0.1130 (4)	0.0500 (7)	
H8	0.9293	0.4971	0.1330	0.060*	
C9	0.8186 (4)	0.5967 (2)	0.0306 (3)	0.0485 (7)	
H9	0.7899	0.5226	−0.0055	0.058*	
C10	1.0386 (3)	0.6850 (3)	0.2567 (2)	0.0498 (7)	
H10	1.0723	0.6038	0.2794	0.060*	
C11	1.0740 (4)	0.7970 (3)	0.3077 (3)	0.0555 (8)	
H11	1.1295	0.7922	0.3660	0.067*	
C12	1.0266 (3)	0.9184 (3)	0.2720 (2)	0.0471 (7)	
H12	1.0515	0.9940	0.3076	0.056*	
C13	1.1578 (3)	1.2130 (2)	0.07154 (19)	0.0303 (5)	
C14	1.0979 (3)	1.2700 (2)	0.17167 (19)	0.0288 (5)	

### Atomic displacement parameters (Å^2^)

**Table:**

	*U*^11^	*U*^22^	*U*^33^	*U*^12^	*U*^13^	*U*^23^
C1	0.0439 (15)	0.0420 (14)	0.0362 (14)	0.0018 (12)	−0.0088 (12)	−0.0036 (12)
Cu1	0.03102 (14)	0.02964 (14)	0.03119 (13)	−0.00071 (10)	−0.00153 (13)	−0.00414 (16)
N1	0.0315 (11)	0.0393 (11)	0.0347 (12)	0.0005 (9)	−0.0041 (9)	−0.0007 (9)
O1	0.0370 (10)	0.0409 (9)	0.0350 (10)	−0.0020 (8)	0.0025 (8)	−0.0107 (8)
C2	0.0555 (19)	0.0558 (17)	0.0346 (15)	0.0010 (14)	−0.0120 (13)	−0.0089 (13)
N2	0.0336 (10)	0.0323 (10)	0.0317 (11)	0.0008 (8)	−0.0013 (9)	−0.0039 (9)
O2	0.0365 (11)	0.0433 (10)	0.0335 (10)	−0.0101 (8)	0.0092 (8)	−0.0118 (9)
C3	0.0500 (18)	0.0539 (17)	0.0376 (16)	−0.0090 (14)	−0.0026 (14)	−0.0157 (13)
O3	0.0382 (10)	0.0329 (9)	0.0361 (10)	−0.0043 (8)	0.0007 (8)	−0.0064 (8)
C4	0.0393 (14)	0.0370 (13)	0.0395 (15)	−0.0068 (11)	0.0064 (11)	−0.0096 (12)
O4	0.0363 (10)	0.0388 (9)	0.0377 (10)	−0.0026 (8)	0.0102 (8)	−0.0030 (8)
C5	0.0320 (13)	0.0337 (13)	0.0307 (13)	−0.0018 (11)	0.0058 (11)	−0.0035 (10)
C6	0.0288 (12)	0.0328 (12)	0.0305 (16)	0.0004 (9)	0.0055 (9)	−0.0016 (9)
C7	0.0375 (14)	0.0414 (15)	0.0424 (15)	0.0040 (12)	0.0059 (12)	0.0067 (12)
C8	0.0599 (17)	0.0306 (12)	0.0593 (18)	0.0031 (10)	0.011 (2)	0.0032 (18)
C9	0.0581 (18)	0.0317 (15)	0.056 (2)	−0.0092 (12)	0.0074 (16)	−0.0072 (13)
C10	0.0491 (18)	0.0495 (18)	0.0509 (19)	0.0092 (13)	0.0012 (14)	0.0111 (14)
C11	0.0519 (19)	0.068 (2)	0.0465 (19)	0.0056 (17)	−0.0099 (15)	0.0107 (17)
C12	0.0499 (17)	0.0496 (16)	0.0417 (16)	0.0011 (13)	−0.0124 (13)	−0.0052 (13)
C13	0.0327 (13)	0.0285 (12)	0.0296 (12)	0.0062 (10)	−0.0010 (11)	0.0017 (10)
C14	0.0293 (12)	0.0297 (12)	0.0274 (12)	0.0011 (10)	−0.0030 (10)	−0.0022 (11)

### Geometric parameters (Å, °)

**Table:**

C1—N2	1.311 (3)	O3—Cu1^ii^	2.3135 (18)
C1—C2	1.403 (4)	C4—C5	1.407 (3)
C1—H1	0.9300	C4—C9	1.428 (4)
Cu1—O2	1.9753 (18)	O4—C13	1.263 (3)
Cu1—O4^i^	1.9973 (19)	O4—Cu1^ii^	1.9973 (19)
Cu1—N2	2.024 (2)	C5—C6	1.428 (3)
Cu1—N1	2.049 (2)	C6—C7	1.414 (3)
Cu1—O1	2.2909 (19)	C7—C10	1.401 (4)
Cu1—O3^i^	2.3135 (18)	C7—C8	1.426 (5)
N1—C12	1.325 (4)	C8—C9	1.350 (6)
N1—C6	1.359 (3)	C8—H8	0.9300
O1—C13	1.247 (3)	C9—H9	0.9300
C2—C3	1.360 (5)	C10—C11	1.363 (4)
C2—H2	0.9300	C10—H10	0.9300
N2—C5	1.356 (3)	C11—C12	1.390 (4)
O2—C14	1.260 (3)	C11—H11	0.9300
C3—C4	1.395 (4)	C12—H12	0.9300
C3—H3	0.9300	C13—C14	1.554 (3)
O3—C14	1.234 (3)		
			
N2—C1—C2	123.5 (2)	C3—C4—C9	124.7 (3)
N2—C1—H1	118.3	C5—C4—C9	118.4 (3)
C2—C1—H1	118.3	C13—O4—Cu1^ii^	118.13 (17)
O2—Cu1—O4^i^	93.34 (8)	N2—C5—C4	122.6 (2)
O2—Cu1—N2	173.31 (8)	N2—C5—C6	117.0 (2)
O4^i^—Cu1—N2	91.68 (9)	C4—C5—C6	120.4 (2)
O2—Cu1—N1	93.68 (8)	N1—C6—C7	123.3 (2)
O4^i^—Cu1—N1	172.68 (8)	N1—C6—C5	116.9 (2)
N2—Cu1—N1	81.49 (9)	C7—C6—C5	119.8 (2)
O2—Cu1—O1	78.18 (7)	C10—C7—C6	116.2 (2)
O4^i^—Cu1—O1	88.46 (7)	C10—C7—C8	125.5 (3)
N2—Cu1—O1	97.55 (7)	C6—C7—C8	118.3 (3)
N1—Cu1—O1	95.01 (8)	C9—C8—C7	121.7 (3)
O2—Cu1—O3^i^	89.80 (7)	C9—C8—H8	119.1
O4^i^—Cu1—O3^i^	77.92 (7)	C7—C8—H8	119.1
N2—Cu1—O3^i^	95.57 (7)	C8—C9—C4	121.3 (3)
N1—Cu1—O3^i^	100.03 (8)	C8—C9—H9	119.3
O1—Cu1—O3^i^	161.33 (6)	C4—C9—H9	119.3
C12—N1—C6	117.9 (2)	C11—C10—C7	120.2 (3)
C12—N1—Cu1	130.30 (19)	C11—C10—H10	119.9
C6—N1—Cu1	111.77 (16)	C7—C10—H10	119.9
C13—O1—Cu1	108.21 (16)	C10—C11—C12	119.6 (3)
C3—C2—C1	118.2 (3)	C10—C11—H11	120.2
C3—C2—H2	120.9	C12—C11—H11	120.2
C1—C2—H2	120.9	N1—C12—C11	122.7 (3)
C1—N2—C5	118.1 (2)	N1—C12—H12	118.6
C1—N2—Cu1	129.22 (18)	C11—C12—H12	118.6
C5—N2—Cu1	112.62 (16)	O1—C13—O4	125.2 (2)
C14—O2—Cu1	118.30 (16)	O1—C13—C14	117.7 (2)
C2—C3—C4	120.6 (3)	O4—C13—C14	117.1 (2)
C2—C3—H3	119.7	O3—C14—O2	124.9 (2)
C4—C3—H3	119.7	O3—C14—C13	118.5 (2)
C14—O3—Cu1^ii^	108.00 (16)	O2—C14—C13	116.6 (2)
C3—C4—C5	116.9 (2)		

Symmetry codes: (i) *x*−1/2, −*y*+5/2, *z*; (ii) *x*+1/2, −*y*+5/2, *z*.

### Hydrogen-bond geometry (Å, °)

**Table:**

*D*—H···*A*	*D*—H	H···*A*	*D*···*A*	*D*—H···*A*
C11—H11···O4^iii^	0.93	2.51	3.416 (4)	166
C9—H9···O1^iv^	0.93	2.49	3.160 (3)	129
C2—H2···O2^v^	0.93	2.52	3.136 (3)	124
C1—H1···O4^i^	0.93	2.56	3.072 (3)	115

Symmetry codes: (iii) −*x*+5/2, *y*−1/2, *z*+1/2; (iv) *x*−1/2, −*y*+3/2, *z*; (v) −*x*+3/2, *y*−1/2, *z*−1/2; (i) *x*−1/2, −*y*+5/2, *z*.
